# Radiological findings of mucormycosis rhinosinusitis among Indian COVID-19 patients during the pandemic second wave

**DOI:** 10.1186/s43163-023-00457-5

**Published:** 2023-06-06

**Authors:** Roger Anthony Manuel, Arun George

**Affiliations:** Department of Radiodiagnosis, St. John’s National Academy of Health Sciences, Bengaluru, India

**Keywords:** Sinonasal mucormycosis, Fungal sinusitis, COVID-19, Rhino-orbital mucormycosis, Rhino-orbitocerebral mucormycosis, Rhinocerebral mucormycosis

## Abstract

**Background:**

Sinonasal mucormycosis is a quickly progressing and lethal fungal disease which showed an increased incidence in COVID-19 patients in the Indian population during the second wave of the pandemic. The objective of this study was to study the various sinus areas affected and the imaging findings of the disease.

**Methods:**

The imaging records of patients with sinonasal mucormycosis during the second wave of the COVID-19 pandemic were reviewed and analysed for whom computed tomography (CT) and/or magnetic resonance imaging (MRI) images had been performed.

**Results:**

Of the 65 patients, 6.1% had single sinus involvement, and 93.9% had multiple sinus involvement, and out of latter, 91.8% had bilateral sinuses affected by the disease process. A total of 49.2% patients with sinus involvement had erosions of the sinus walls. A total of 35.4% patients had only sinonasal mucormycosis, 38.5% patients had rhino-orbital mucormycosis, 4.6% patients had rhino-cerebral mucormycosis and 16.9% patients had rhino-orbitocerebral mucormycosis. The pterygopalatine fossa was affected in 26.2% patients. A total of 9.2% patients had cavernous sinus thrombosis. A total of 12% of the cases had infarction in the cerebral hemispheres.

**Conclusion:**

In a setting of sinonasal mucormycosis, especially in the immuno-compromised and with those infected with COVID-19, cross-sectional imaging can assess the presence and extent of the disease and helps plan its medical and surgical management.

**Supplementary Information:**

The online version contains supplementary material available at 10.1186/s43163-023-00457-5.

## Background

Mucormycosis is a quickly progressing and lethal disease caused by the fungal species *Rhizomucor* and *Mucor* that usually begins in the nasal cavity and paranasal sinuses [1]. The pathogen infects immunologically compromised patients like uncontrolled diabetics [1].

The disease is noted to invade blood vessels and often causes vascular invasion into the orbit. Infection spreads to other sites including the orbits and the brain. The former occurs via the nasolacrimal duct and medial orbital wall. The latter happens due to invasion and thrombosis of the adjacent blood vessels resulting infarction to the supplied tissues, here being the brain [2].

Survival of those infected patients depends on early diagnosis, aggressive surgical debridement, parenteral antifungal drugs like amphotericin and management of the patient’s underlying illness like diabetes for example [2].

COVID-19 (coronavirus disease-2019) is an infectious disease due to the pathogen severe acute respiratory syndrome coronavirus 2 (SARS-CoV-2) [3]. Infection can vary from an asymptomatic state to a serious disease state, with death being the common outcome of acute lung injury [3].

The prevalence of rhino-orbital mucormycosis in Indians with coronavirus disease 2019 (COVID-19) increased during the second wave of the pandemic. Diabetes mellitus (DM), a cause for immune-compromise, is an independent risk factor for severe COVID-19 and sinonasal mucormycosis [4–6].

The clinical presentation of mucormycosis includes rhino-orbital-cerebral, pulmonary, cutaneous, gastrointestinal and disseminated varieties [7].

Early detection and accurate diagnosis help in the proper assessment of the disease process and that in turn the treatment which is aimed at reduced morbidity and mortality. In this study, we aimed at studying the various common areas involved by sinonasal mucormycosis in a background of COVID-19 during the second wave.

## Main text

### Objective

To describe the cross-sectional (CT & MR) imaging findings of sinonasal mucormycosis in COVID-19 patients.

## Methods

This retrospective study which was approved by the ethics board of our institution included 65 COVID-19 Indian patients, who were being treated for the same at St. John’s Medical College Hospital during the second wave of the pandemic, i.e. from March 2021 to July 2021 (for a period of 5 months), and had subsequently developed sinonasal mucormycosis.

The inclusion criteria were as follows:

1. Patients both male and female between 18 and 60 years of age who were tested for COVID-19 and subsequently developed clinical symptoms and signs of sinonasal mucormycosis and were thus investigated via cross-sectional imaging, i.e. computed tomography and magnetic resonance imaging (plain and contrast). The diagnosis of mucormycosis was confirmed by tissue biopsy.

The exclusion criteria were as follows:

1. Patients who had pre-existing sinonasal mucormycosis prior to the diagnosis of COVID-19 infection
2. Motion artefacts in the imaging of those patients which precluded optimal observation and accurate diagnosis

Questionnaires for the presence of allergy to intravenous contrast agents prior to the imaging investigation (CT and MRI), and for the presence of MR compatible implants and pacemakers before the MR study, were filled and recorded in accordance with patient safety norms and rules. Only those who were deemed fit and safe for the investigation were imaged.

For obtaining CT images, a GE 128 slice CT scanner was employed which detected mainly bony changes like erosions and bony destruction. Plain images of the head, paranasal sinuses and orbits were obtained at 5-mm slice thickness, and reconstruction interval of 0.6 mm was employed. Contrast images were obtained with an injection bolus of 100 cc of iohexol intravenously, and images were taken 50 s post injection of the contrast agent. Using multi-plane reformation (MPR) technique, the images were analysed further in the axial, sagittal and coronal planes at the PACS workstation.

For obtaining MR images, GE 3.0 T MRI scanner was used which detected soft tissue involvement and orbital and brain extension. Gadolinium was injected via the intravenous route for contrast studies. The sequences performed were axial DWI and axial T2 FLAIR. SAG T1 FLAIR, axial SWAN, COR T2 FLAIR and dedicated orbit sequences (COR and SAG T2 orbital sections) were also done where necessary.

The images obtained were reviewed independently by two radiologists with at least 8 years of experience, and the findings were compared and analysed.

The parameters that were looked into were the presence or absence of the following:

1. Sinonasal involvement
2. Single or multiple paranasal sinus involvement
3. Bilateral paranasal sinus involvement
4. Bony erosion of the wall of the paranasal sinuses
5. Extra-sinus spread/invasion of the following:
  a Orbit
  b Cranium
  c Pterygopalatine fossa
6. The presence of cavernous sinus thrombosis
7. Infarction of cerebral parenchyma

Also, the number of patients with only sinonasal, sinonasal and orbital (rhino-orbital mucormycosis), sinonasal and cerebral (rhino-cerebral) and sinonasal, orbital and cerebral (rhino-orbitocerebral mucormycosis) involvement was documented.

Additionally, the levels of HbA1c were also checked for the patients included in the study. Patients with other immunosuppressive states such as renal failure and cytotoxic medication were not included in the study.

## Results

Of the 65 patients, 11 were females, and 54 were males, and the majority (95%) was above 40 years of age. Sixty-three (97%) of the 65 patients had elevated HbA1c levels and were diagnosed with diabetes mellitus.

Of the 65 patients, 4 (6.1%) had single sinus involvement, and 61 (93.9%) had multiple sinus involvement, and out of latter, 56 (91.8%) had bilateral sinuses affected by the disease process. Thirty-two (49.2%) patients with sinus involvement had erosions of the sinus walls.

**Fig. 1:**
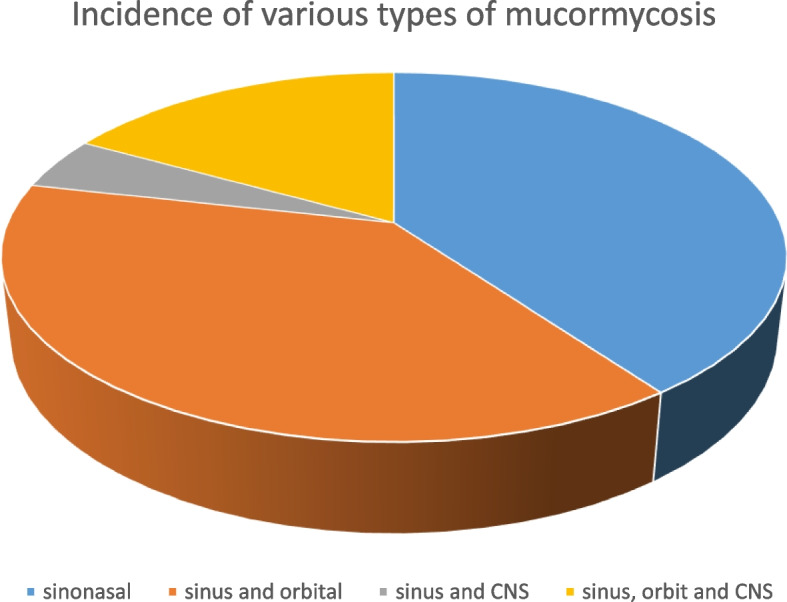
Showing the incidence of the various types of mucormycosis in the study

Twenty-six (35.4%) patients had only sinonasal involvement. Twenty-five (38.5%) patients had sinus and orbital involvement. Three (4.6%) patients had sinus and central nervous system involvement. Eleven (16.9%) patients had their sinuses, orbits and the central nervous system affected. There were no patients in this study with only orbital or central nervous system involvement as shown in Fig. 1.

In 17 (26.2%) patients, the pterygopalatine fossa was affected. Six (9.2%) patients had cavernous sinus thrombosis. Eight of the 14 patients with cerebral involvement (57%) and 12% of the cases in total had infarction in the cerebral hemispheres.

None of the participants in the study had pulmonary thromboembolism during the duration of the study.

**Fig. 2:**
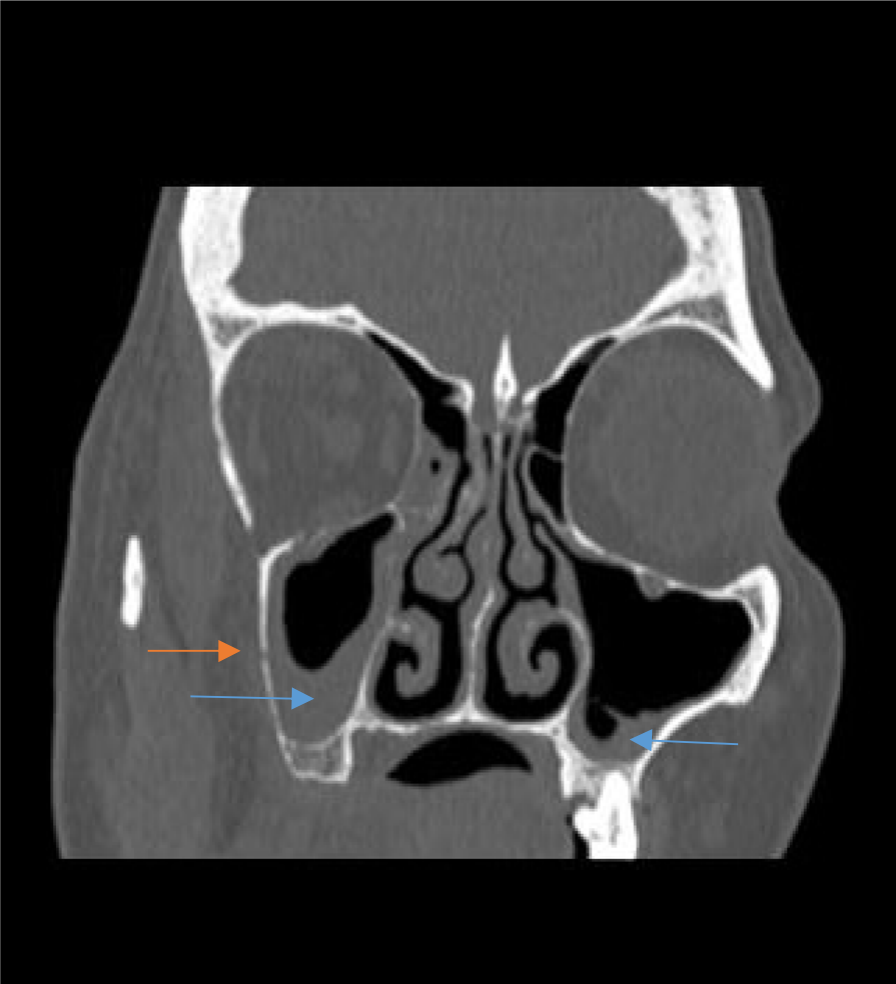
Coronal image of a plain CT of the paranasal sinuses in a patient with sinonasal mucormycosis showing mucosal thickening (blue arrows) in bilateral maxillary sinuses (R > L) with rarefaction and focal erosions (red arrow) in the wall of the right side

The involved paranasal sinuses showed mucosal thickening, which ranged from mild to severe and was even polypoidal in a few cases. Post contrast administration, the mucosal thickening was not seen to enhance. There was rarefaction and erosion of the sinus walls on CT imaging (Fig. 2). Depending on the location and the sinuses involved, the spread of the disease process was thus noted.

Orbital involvement was seen in cases with ethmoid sinus involvement with the disease gaining access to the orbit via the medial wall which was eroded. Orbital involvement ranged from fat stranding, extra-occular muscle necrosis to collections and abscess formation.

**Fig. 3:**
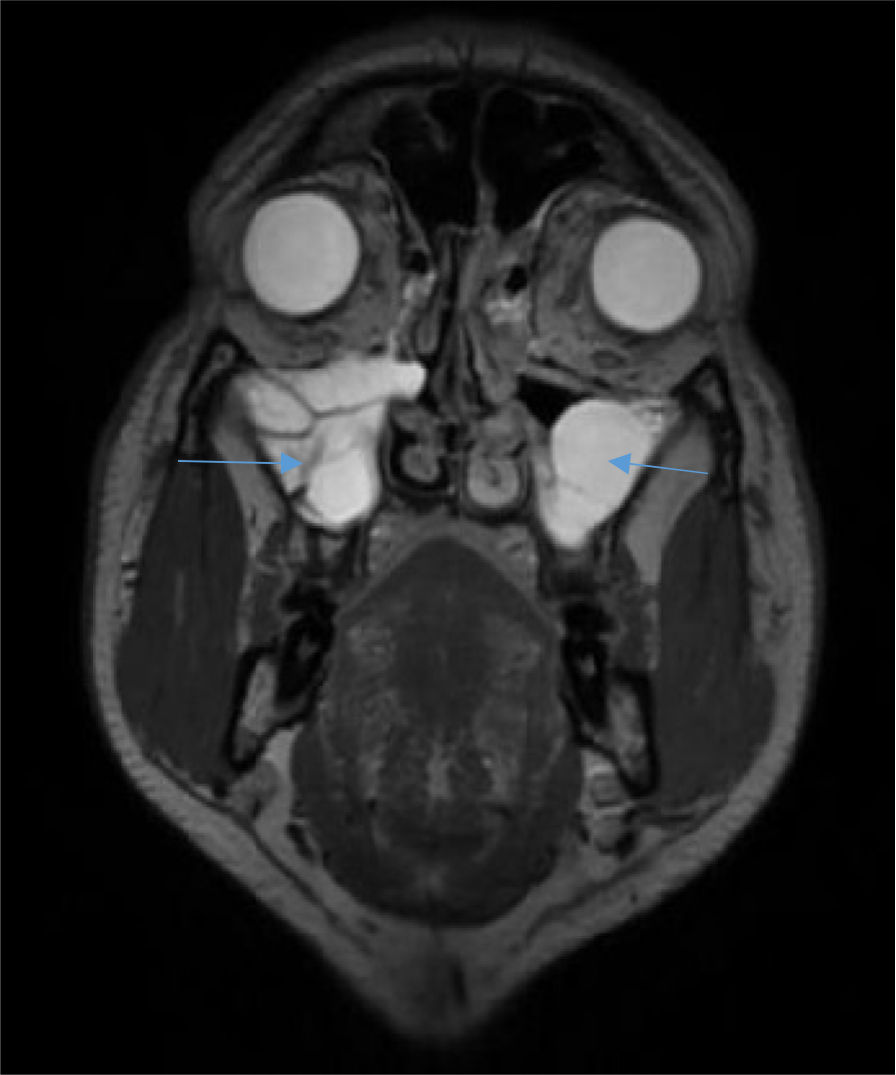
Coronal section of T2-weighted image of a plain MRI of the paranasal sinuses in a patient with sinonasal mucormycosis showing T2 hyperintense mucosal thickening in bilateral maxillary sinuses (blue arrows)

From the maxillary sinus, the disease was noted to spread to the pterygopalatine fossa, pterygoid muscles and the foramen rotundum. On MR imaging, the mucosal thickening was seen to be iso-intense on T1-weighted imaging and hyperintense on T2-weighted imaging (Fig. 3). In those cases that MR contrast study was conducted, the mucosal thickening was noted to be non-enhancing. In cases with abscess formation, diffusion restriction was seen in the affected areas. Fat stranding and collections were also detected in the orbit (intra and extra-conal spaces) and in the pterygopalatine fossa and muscles in the affected cases.

**Fig. 4:**
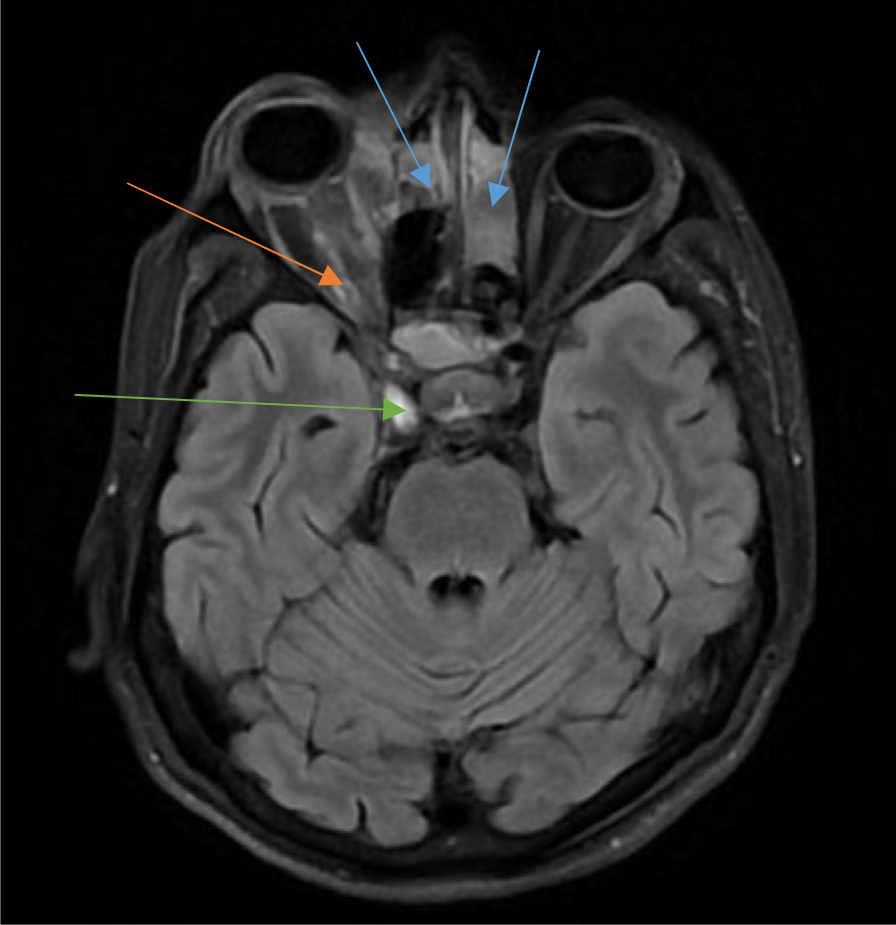
Axial section of contrast-enhanced T1-weighted image of an MRI of the brain in a patient with rhino-orbitocerebral mucormycosis showing heterogeneously enhancing mucosal thickening in bilateral ethmoid sinuses (blue arrows) with enhancing soft tissue surrounding the intra-orbital segment of the right optic nerve (red arrow). Also noted is lack of flow void in the right cavernous sinus indicating thrombosis (green arrow)

A non-enhancing filling defect in contrast-enhanced MRI T1-weighted images was seen in cases in which the cavernous sinus showed thrombosis as a complication (Fig. 4).

Of the 65 patients, 63 (97%) of the patients in our study were diagnosed with diabetes mellitus via elevated HbA1c levels. Out of these, 39 (60%) patients were found to have HbA1c levels of 9 and above, and these patients were found to have more extensive involvement, namely bilateral sinuses affected with bony erosions, necrosis and spread of infection to the orbits and the brain. The diagnosis of mucormycosis in the patients included in the study was confirmed by tissue biopsy.

## Discussion

The prevalence of mucormycosis in the Indian population is higher than the rest of the global population. Chander et al. in 2018 proposed that India has 80 times the universal average of mucormycosis prevalence, with an estimate of 0.14 cases per 1000 diabetic persons [8].

The estimates of the Leading International Fungal Education (LIFE) organisation states that the prevalence of mucormycosis worldwide ranged between 0.6 and 3 cases per million people, but in India, it was 140 cases per million people [9].

According to Sushen Kumar et al., the radiological findings of mucormycosis in post-COVID-19 patients show different forms of involvement and imaging features, and CT and MR were useful for assessing the disease spread and invasion into nearby anatomical structures [10].

Sandeep Singh et al. in their work studied the aggressive necrosis caused by rhino-orbital mucormycosis [11].

Marina Saldanha et al. reported a case of COVID-19 patient with orbital apex syndrome caused by sinonasal mucormycosis and who required endoscopic surgery, thus asserting the need for speedy detection and surgical and medical treatment [12].

Arora et al. in their work on fungal sinusitis found that mucormycosis involving the paranasal sinuses also affected the orbit, palate and the brain to varying degrees [13].

The pathogen causes aggressive sinonasal and orbital findings on imaging as proven by Herrera et al. [14]. Mohindra et al. have shown that MRI can detect cavernous sinus invasion and vascular complications such as thrombosis and ischemia [15]. Middlebrooks et al. had shown that CT can assess for acute invasive fungal sinusitis by detecting bone dehiscence, orbital invasion, septal erosions and periantral fat, pterygopalatine fossa, nasolacrimal duct and lacrimal sac involvement [16].

Jacob et al. also found that rhino-cerebral mucormycosis spreads from the paranasal sinuses either directly or via the orbits involving them first along the route of spread to the cranial cavity [17].

In our study, which involved 65 patients, all of which had sinonasal mucormycosis, the disease process primarily initiated in the paranasal sinuses which later spread to the pterygopalatine fossa orbits and to the intra-cranial compartment. The cavernous sinus was noted to be thrombosed in a few patients.

Sinonasal mucormycosis was seen to be the most common type of mucormycosis in our study, which was followed by rhino-orbital and rhino-orbitocerebral mucormycosis. Rhino-cerebral mucormycosis was the least common type.

**Fig. 5:**
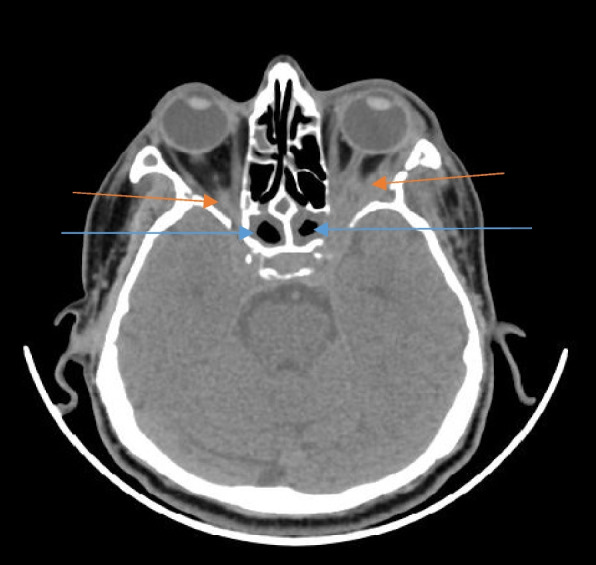
Axial section of a plain CT of the brain in a patient with rhino-orbital mucormycosis showing mucosal thickening in bilateral sphenoid sinuses (blue arrows) with spread into the intra-conal spaces of bilateral orbits as seen by involvement of intra-orbital segments of bilateral optic nerves with surrounding soft tissue and fat stranding (red arrows)

CT detected orbital involvement and the extent of the disease process in the intra-conal and extra-conal spaces (Fig. 5). Even the presence of only fat stranding without any significant change in the attenuation or morphology of the tissues was seen to be a good indicator of early spread of the disease and a predictor of the subsequent involvement of the affected regions.

**Fig. 6:**
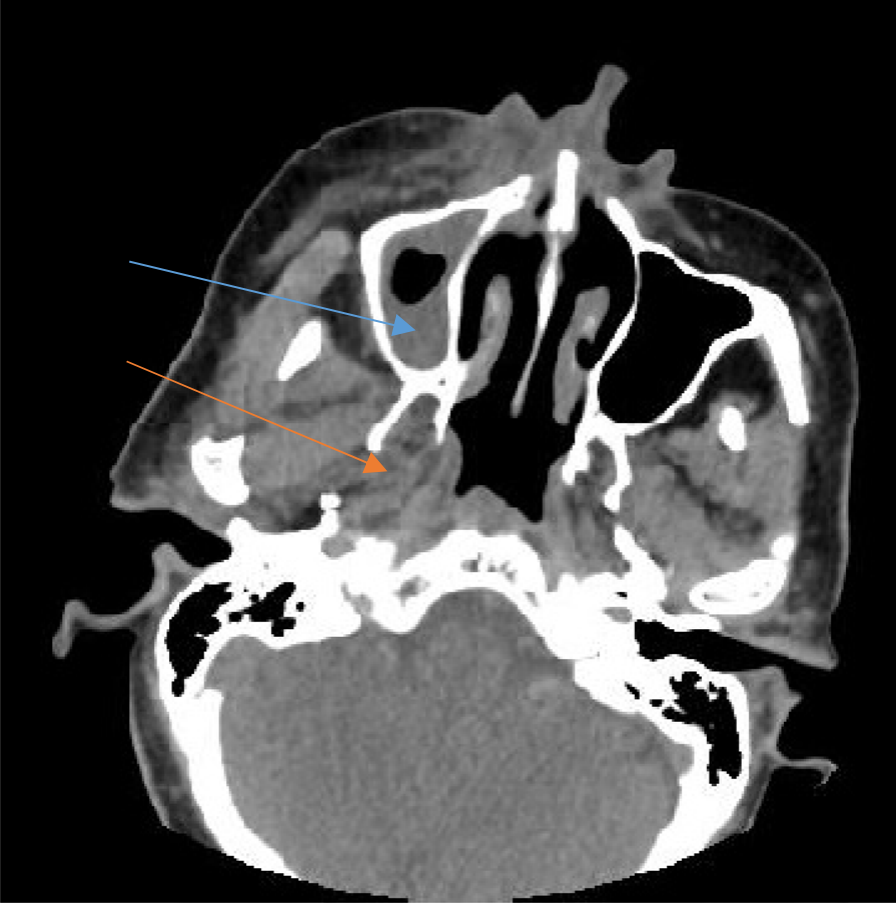
Axial section of plain CT of the brain in a patient with sinonasal mucormycosis showing mucosal thickening of the right maxillary sinus (blue arrow) with involvement of the right pterygopalatine fossa (red arrow)

The disease process after involving the sinuses whether single or multiple spread into the adjacent areas, namely the pterygopalatine fossa (Fig. 6) and the orbits. Rarefaction and erosion of the sinus walls were seen to be a common sign of bony involvement on CT imaging.

**Fig. 7:**
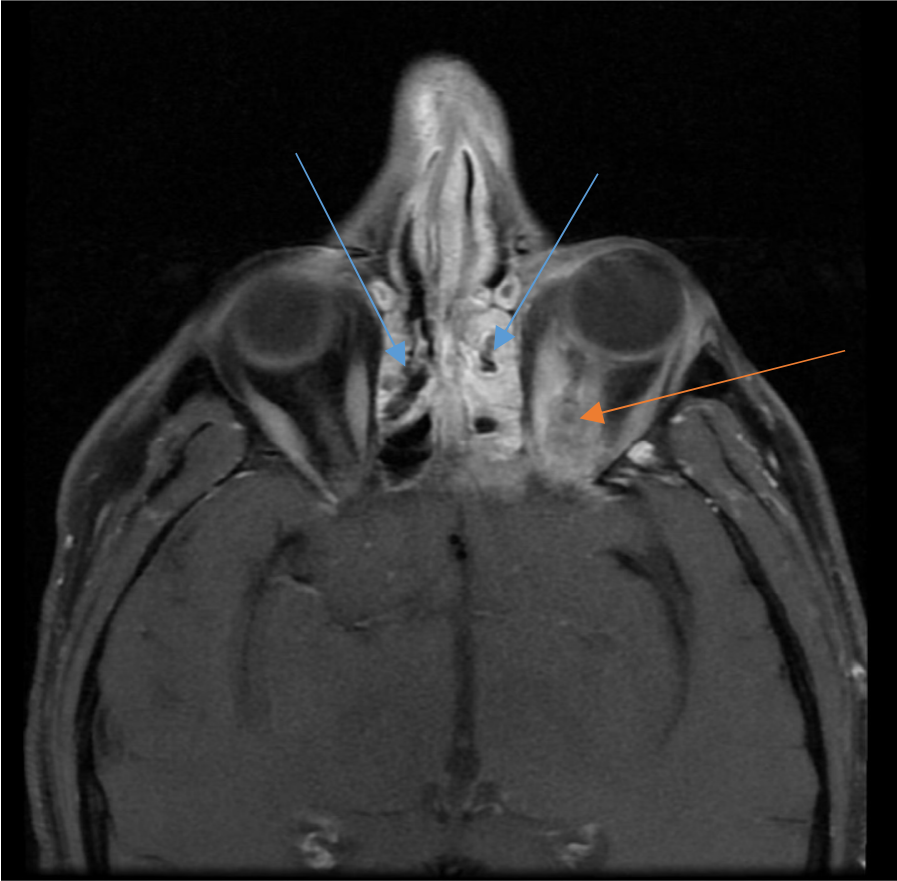
Axial section of contrast-enhanced T1-weighted image of an MRI of the brain in a patient with rhino-orbital mucormycosis showing enhancing mucosal thickening of bilateral ethmoid sinuses (blue arrows) along with enhancing soft tissue around the intra-orbital segment of the left optic nerve in the intra-conal compartment and surrounding fat stranding (red arrow)

MR imaging is useful in detecting the soft tissue involvement in the face and orbits (Fig. 7). Diffusion restriction detected necrosed tissues and abscesses/collections in patients without the use of iodinated contrast especially with chronic kidney disease (secondary to diabetic nephropathy).

The black turbinate sign was seen in those cases with necrosed nasal tissue and the turbinates where there was lack of enhancement post contrast administration.

**Fig. 8:**
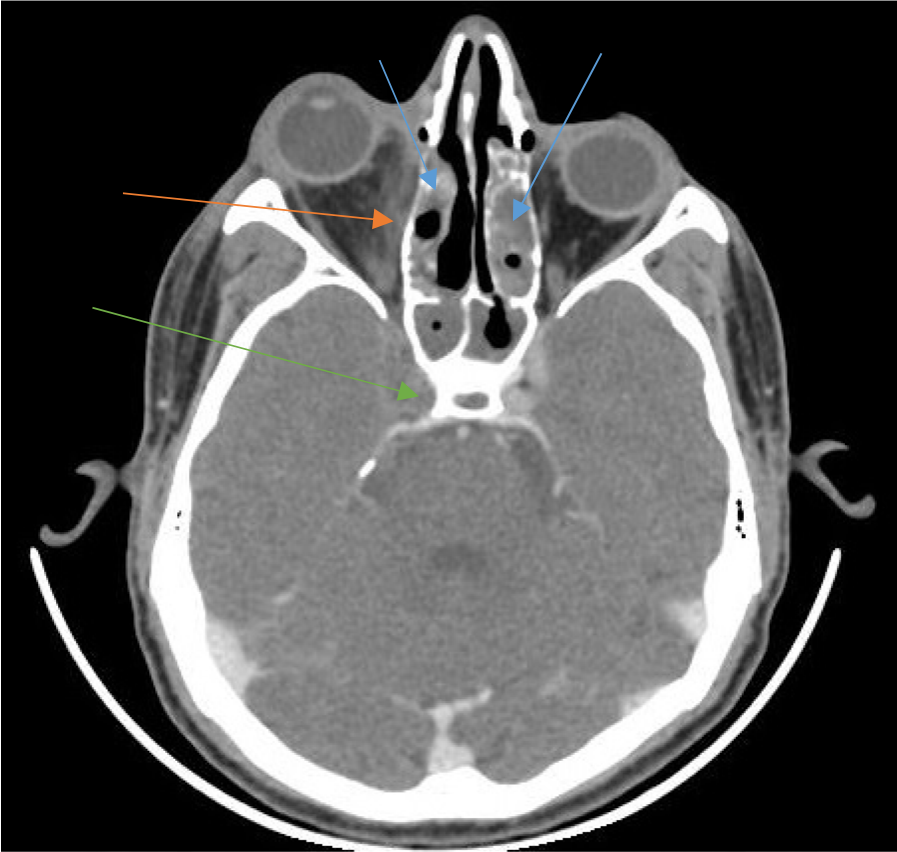
Axial section of contrast-enhanced CT of the brain in a patient with rhino-orbitocerebral mucormycosis showing non-enhancing mucosal thickening in bilateral ethmoid sinuses (blue arrows) with fat stranding in the medial aspect of the right orbit (red arrow). The cavernous segment of the right internal carotid artery is not opacified by the contrast as compared to the left side, suggestive of thrombosis (green arrow)

**Fig. 9:**
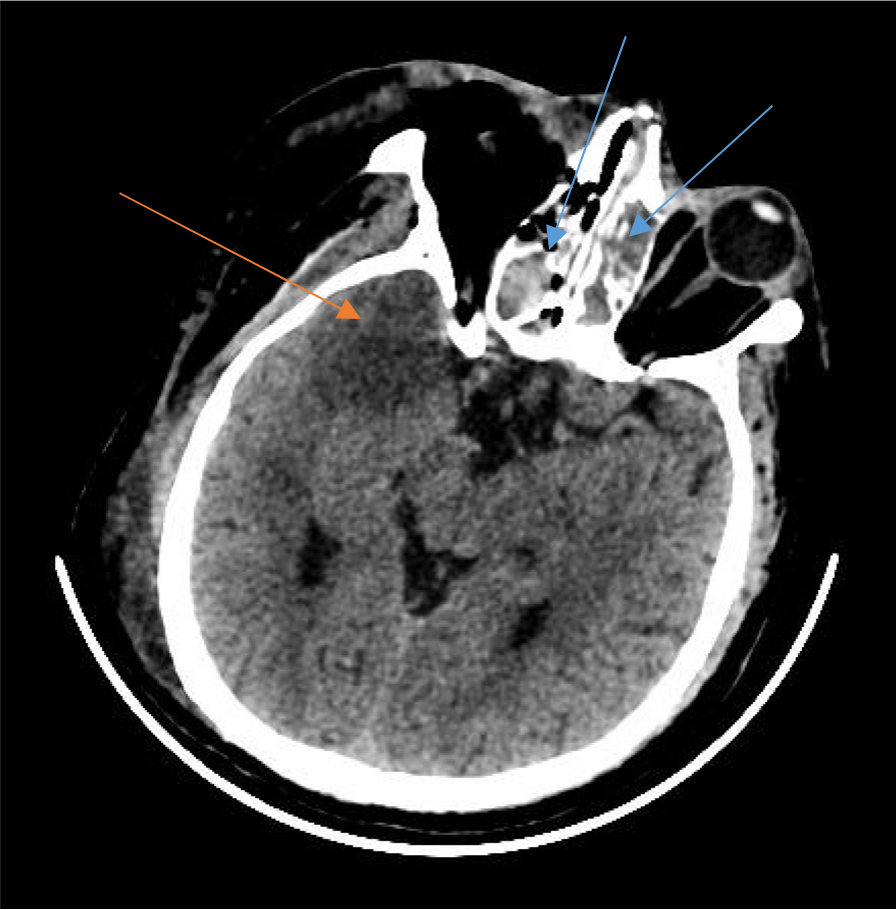
Axial section of plain CT of the brain in a patient with rhino-cerebral mucormycosis showing hyperdense mucosal thickening of bilateral ethmoid sinuses (blue arrows) and a wedge-shaped hypodense area in the right temporal lobe suggestive of acute infarct of the right middle cerebral artery territory (red arrow)

CT also picked up thrombosis of internal carotid and middle cerebral arteries (Fig. 8) causing infarcts of the cerebral parenchyma (Fig. 9).

Cavernous sinus thrombosis is also a feature of the thrombotic nature of the disease process. On surgical exploration of the patients, the involvement of the affected areas and the extent of the disease process were confirmed.

Those patients with extensive involvement were found to have more morbidity, requirement of extensive surgical procedures (orbital exenteration) and longer recovery time.

Both CT and MR are useful in the preoperative assessment of the disease and in the post-operative period as well for searching for residual disease and recurrence. With information on bony details in CT and the extent of soft tissue involvement in MR, both modalities are complementary to each other in the evaluation of patients with sinonasal mucormycosis.

The high incidence of sinonasal mucormycosis in the Indian population during the second wave of the COVID pandemic could be due to the higher incidence of diabetes mellitus.

### Limitations of the study

Both CT and MR imaging performed was not performed for all the patients, due to their financial constraints, and so the soft tissue involvement (orbital and cerebral) may have been under-reported at the time of the investigation.

## Conclusions

After taking into consideration the findings of our study, the usefulness of CT and MR imaging in the detection and diagnosis of sinonasal mucormycosis along with its spread to the orbit and the intra-cranial compartment is confirmed. Therefore, in a setting of sinonasal mucormycosis, especially in the immuno-compromised, diabetic and with those infected with COVID-19, cross-sectional imaging can assess the presence and extent of the disease and helps plan its medical and surgical management.

## Supplementary Information

**Additional file 1.** Sinonasal mucormycocsis supplementary

## Data Availability

The datasets used and/or analysed during the current study are available from the corresponding author on reasonable request.

## Abbreviations

PACS: Picture archiving and communication system
CT: Computed tomography
MR: Magnetic resonance
SAG: Sagittal
COR: Coronal
DWI: Diffusion-weighted imaging
FLAIR: Fluid-attenuated inversion recovery
SWAN: Susceptibility-weighted angiography
MPR: Multi-plane projection
HbA1c: Glycated haemoglobin
RAM: Roger Anthony Manuel
AG: Arun George
