# Supplementary material for: Radiological findings of mucormycosis rhinosinusitis among Indian COVID-19 patients during the pandemic second wave
Source: Egypt J Otolaryngol. 2023 Jun 6;39(1):94. doi: 10.1186/s43163-023-00457-5 (PMC10242232; doi:10.1186/s43163-023-00457-5)
Supplement: Supplementary file 1 — Additional file 1. Sinonasal mucormycocsis supplementary [file 43163_2023_457_MOESM1_ESM.docx]

**SUPPLEMENTARY**

**Radiological findings of mucormycosis rhinosinusitis among Indian COVID-19 patients during the pandemic second wave**

Primary Author:

Dr. Roger Anthony Manuel (RAM)

MD. Radiodiagnosis

Email: [manuelroger33@yahoo.in](mailto:manuelroger33@yahoo.in)

Phone number: +91-9945211924

Secondary Author:

Dr. Arun George (AG)

MD. Radiodiagnosis

Email: [arungeorge@email.com](mailto:arungeorge@email.com)

Institution name: St. John’s National Academy of Health Sciences, Bengaluru, India

**Authors’ contribution:**

RAM was involved in recruiting the patients, studying the images, conducting the statistical analysis including the analysis and interpretation of the data and was a major contributor in writing and preparing the manuscript.

AG was involved in studying the images and advising on statistical analysis.

All the authors read and approved the final manuscript.

**Ethics approval and consent to participate:** Approval from the **Institutional Ethics Committee of St. John’s Medical College and Hospital** was obtained for the study prior to commencement of the study and it was assigned the number **IEC/723/2022.** Written consent of the patients to participate was taken prior to including them in the study.

**Consent for publication**: Written consent for publication was obtained from the patients participating in the study after informing them about the purpose of the study.
